# The effects of the mother’s ADHD on her parenting role

**DOI:** 10.1192/j.eurpsy.2023.553

**Published:** 2023-07-19

**Authors:** C. Moutafi

**Affiliations:** private practice, Athens, Greece; private practice, Athens, Greece

## Abstract

**Introduction:**

Attention Deficit Hyperactivity Disorder (ADHD) is the second most common psychiatric disorder in childhood, affecting 5-7% of the child population. The same disorder in adults is less documented and is estimated at 2.5% of the population. The mothers of children with ADHD are 24 times more likely to develop ADHD than mothers of children without ADHD. About 17% of mothers of children with ADHD meet criteria for the disorder themselves. The core symptoms of ADHD are inattention, hyperactivity and impulsivity.

**Objectives:**

The objective of this e-poster is to describe the effects of the mother’s ADHD on her parenting role

**Methods:**

The current poster is based on the bibliographic reviews of papers via the ‘PubMed’ search engines.

**Results:**

In some studies mothers with ADHD may use ineffective discipline methods in order to limit their children.The mothers with ADHD often have difficulties with executive functions such as planning, organizing and implementing goals and with self-control. Characteristically, in a study with a sample of mothers with children aged 3-6 years were observed difficulties in defining the boundaries of the children, unstable behavior and low self-esteem. Additionally, mothers with ADHD may have difficulty forming and maintaining social relationships and this have a negative effect on children’s social skills. The maternal role is influenced by when the mother suffers from depression, anxiety, uses alcohol or psychotropic substances. In addition, her role is influenced by when there are problems in the marital relationship or she is a single parent. The reactions of the mother with ADHD may be influenced not only by whether she has ADHD but also by whether or not her child has been diagnosed with ADHD. The mother with ADHD and child with ADHD has to deal not only with her own symptoms but also with her child’s symptoms. The coexistence of the specific disorder in both may cause increased levels of stress to the mother. Nevertheless, some research reports that these mothers show empathy towards their children, i.e. they have a more positive and supportive behavior, they are more protective, less irritable and less frustrating (similarity-fit hypothesis). In other studies mothers with ADHD worsen their children’s symptoms with their behavior (similarity-misfit hypothesis). There is a significant correlation between the mother’s ADHD and the child’s emotional, behavioral and social functioning.

**Conclusions:**

In conclusion, mothers with ADHD may experience difficulties in all developmental stages of their children. Therefore, the treatment of the disorder (medication and cognitive-behavioral psychotherapy) is necessary in order to improve the mothers’ symptoms, the mental condition of their children and the family’s quality of life.

**Disclosure of Interest:**

None Declared

